# Differences between Belgian and Brazilian Group A Streptococcus Epidemiologic Landscape

**DOI:** 10.1371/journal.pone.0000010

**Published:** 2006-12-20

**Authors:** Pierre Robert Smeesters, Anne Vergison, Dioclécio Campos, Eurico de Aguiar, Veronique Yvette Miendje Deyi, Laurence Van Melderen

**Affiliations:** 1 Infectious Diseases Department, Hôpital Universitaire des Enfants Reine Fabiola, Université Libre de Bruxelles Brussels, Belgium; 2 Laboratoire de Génétique des Procaryotes, Institut de Biologie et de Médecine Moléculaires, Université Libre de Bruxelles Brussels, Belgium; 3 Pediatric Department, Hospital Universitário de Brasília, Universidade de Brasília Brasília, Brazil; 4 Epidemiology and Infection Control Unit, Hôpital Universitaire des Enfants Reine Fabiola, Université Libre de Bruxelles Brussels, Belgium; 5 Microbiology Department, Hospital Regional da Asa Sul Brasília, Brazil; 6 Microbiology Department, Centre Hospitalier Universitaire Brugmann, Université Libre de Bruxelles Brussels, Belgium; Medical University of South Carolina, United States of America

## Abstract

**Background:**

Group A Streptococcus (GAS) clinical and molecular epidemiology varies with location and time. These differences are not or are poorly understood.

**Methods and Findings:**

We prospectively studied the epidemiology of GAS infections among children in outpatient hospital clinics in Brussels (Belgium) and Brasília (Brazil). Clinical questionnaires were filled out and microbiological sampling was performed. GAS isolates were *emm*-typed according to the Center for Disease Control protocol. *emm* pattern was predicted for each isolate. 334 GAS isolates were recovered from 706 children. Skin infections were frequent in Brasília (48% of the GAS infections), whereas pharyngitis were predominant (88%) in Brussels. The mean age of children with GAS pharyngitis in Brussels was lower than in Brasília (65/92 months, p<0.001). *emm*-typing revealed striking differences between Brazilian and Belgian GAS isolates. While 20 distinct *emm*-types were identified among 200 Belgian isolates, 48 were found among 128 Brazilian isolates. Belgian isolates belong mainly to *emm* pattern A–C (55%) and E (42.5%) while *emm* pattern E (51.5%) and D (36%) were predominant in Brasília. In Brasília, *emm* pattern D isolates were recovered from 18.5% of the pharyngitis, although this *emm* pattern is supposed to have a skin tropism. By contrast, A–C pattern isolates were unfrequently recovered in a region where rheumatic fever is still highly prevalent.

**Conclusions:**

Epidemiologic features of GAS from a pediatric population were very different in an industrialised country and a low incomes region, not only in term of clinical presentation, but also in terms of genetic diversity and distribution of *emm* patterns. These differences should be taken into account for designing treatment guidelines and vaccine strategies.

## Introduction

The incidence of Group A Streptococcus (GAS) diseases vary with location and time. Rheumatic fever is still a major cause of cardiovascular morbidity and mortality in developing countries [1], [2], while it has become uncommon in the industrialized world. Pharyngitis occurs predominantly during the cold season in temperate climates and is less frequent in tropical regions [3]. By contrast, skin infections are described at a much higher rate in developing countries than in rich western nations [3], [4]. These epidemiological differences are not or poorly understood.

Molecular GAS typing (*emm* typing) is based on the 5′ end sequence of the *emm* gene that encodes the hyper variable amino-terminal part of the M protein. This surface protein is a major virulence factor and acts as a protective antigen [5]. Currently, more than 170 recognized *emm*-types, constituting more than 750 sub-types, have been described (http://www.cdc.gov/ncidod/biotech/strep/emmtypes.htm). Molecular epidemiological data are available for Western countries where a limited number of specific *emm*-types are predominant. Although few comprehensive and prospective studies have been performed in developing countries [1], a high diversity of *emm*-types has been described in certain locations [6]–[9].

The *emm* pattern serves as a genotypic marker to classify GAS strains according to their tissue tropism (skin or throat). It is based on the organization of *emm* and *emm*-like genes located in the *mga* locus [10], [11]. The *emm* pattern A–C strains are usually recovered from the throat isolates, whereas strains presenting an *emm* pattern D are most often isolated from the skin. “Generalist” *emm* pattern E strains are associated with both tissue sites. Rheumatic fever has been historically associated with strains belonging to *emm* pattern A–C [12]. However, the correlation between *emm* pattern and site of isolation has not always been confirmed in developing countries, where rheumatic fever is still endemic [9], [11]. In Nepal, for instance, A–C strains were preferentially recovered from skin infections [9]. These data indicate no definitive association between GAS *emm* pattern groups and the site of infection and/or the ability to cause a specific disease.

These epidemiological differences prompted us to conduct a comparative study between an industrialized country (Belgium) and a developing country (Brazil).

## Results

### 1) Clinical Epidemiology

706 children (360 in Brussels, 346 in Brasília) with various streptococcal manifestations were included in the study. No significant difference in the mean number of children under the same roof was observed between Brasília (2.2 children) and Brussels (2 children). 334 GAS isolates (204 in Brussels, 161 of which were reported from the laboratory and clinical data completed retrospectively; and 130 in Brasília) were recovered from the 706 children. In Brussels, 94% of the GAS isolates were recovered from the throat or external ear (pharyngitis, scarlet fever and otitis) while cutaneous infections were rare (3%). We observed two GAS invasive infections. In Brasília, GAS were isolated equally from cutaneous infections and pharyngitis. There were 5 GAS invasive infections and 1 sequela (post-streptococcal glomerulonephritis).

#### Risk factors for pharyngitis and impetigo

**Figure 1:**
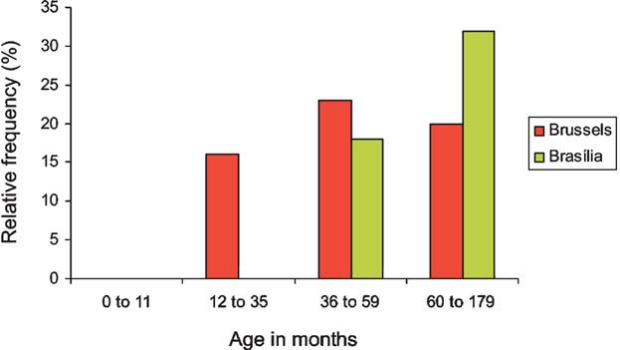
Distribution of GAS pharyngitis according to age in Brussels and Brasília. Percentage of positive GAS throat culture for different age categories of children with pharyngitis. p = 0,003 for the comparison between the three age groups in Brasília.

The percentage of clinically diagnosed pharyngitis associated with GAS positive culture was higher in Brasília (26%) than in Brussels (20%) (p = 0,13). In Brasília, the percentage of positive culture varied with the age of children: 32%, 18% and 0% of the children older than 59 months, from 36 to 59 month old and under 36 month old presented a GAS pharyngitis respectively (p = 0.003) (Figure 1). In Brussels, the prevalence of GAS pharyngitis was unchanged from 15 month old to 13 year old. The mean age of children with GAS pharyngitis was 7.6 year old (ranging from 38 to 153 months) in Brasília and 5.4 year old (ranging from 15 to 150 months) in Brussels (*p*<0.001). Nutrition indices, crowding, sex or previous antibiotherapy did not constitute a risk for GAS pharyngitis in both Brussels and Brasília.

61% of impetigo were associated with a GAS positive culture in Brasília. These streptococcal skin infections were not related to age, sex, crowding, nutrition indices and previous antibiotic treatment during the last six months. The small number of impetigo in Brussels did not allow any statistical analyses.

### 2) Molecular Epidemiology

#### *emm*-types and *emm*-patterns distribution

**Figure 2:**
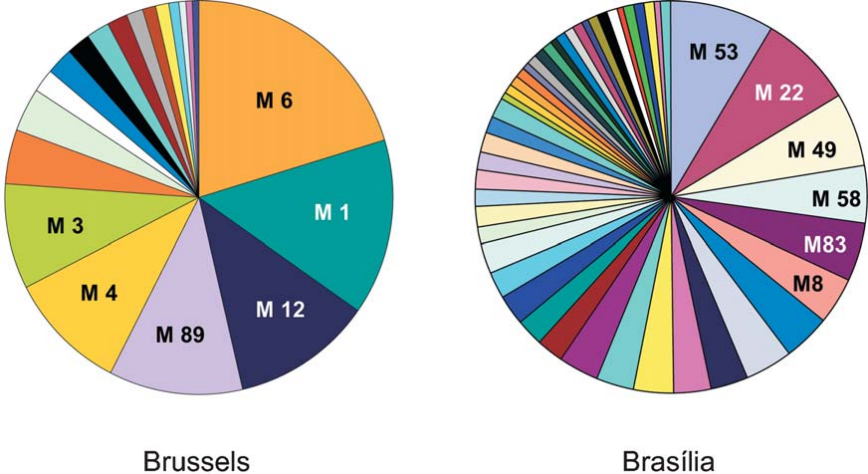
*emm*-types distribution in Brussels and Brasília. The distribution of the *emm*-types is oligoclonal in Brussels (20 *emm*-types among 200 isolates) and polyclonal in Brasília (48 *emm*-types among 128 isolates). Moreover, the *emm*-types involved were different.

The *emm*-type was successfully determined for 200 of the 204 Belgian isolates and 128 of the 130 Brazilian ones. Among the 200 isolates recovered in Brussels, 20 different *emm*-types were identified (figure 2 and table S1). A predominance of 6 *emm*-types, which accounted for 75% of the isolates, was observed in Brussels (*emm* 6 (20%), *emm* 1 (14%), *emm* 12 (12%), *emm* 89 (11%), *emm* 4 (11%) and *emm* 3 (8%)). 55% and 42.5% of the GAS isolates were classified into *emm* patterns A–C and E respectively, whereas only 0.5% presented an *emm* pattern D.

Among the 128 isolates recovered in Brasília which were typed, 48 different *emm*-types were identified (figure 2 and table S2). None of these *emm*-type was predominant. The most frequent *emm*-type (*emm* 53) represented only 8.5% of the Brazilian isolates. The 6 most prevalent *emm*-types (*emm* 53, *emm* 22, *emm* 49, *emm* 58, *emm* 83, *emm* 8) among the Brazilian GAS isolates accounted for only 36% of the total number of GAS isolates and nearly half of the *emm*-types (22/48) were represented by a single isolate. *emm* patterns E, D and A-C accounted for 51.5%, 36% and 9.5% of the Brazilian isolates respectively.

15 *emm*-types (*emm* 1, *emm* 2, *emm* 3, *emm* 4, *emm* 6, *emm* 11, *emm* 12, *emm* 28, *emm* 44/61, *emm* 58, *emm* 64, *emm* 75, *emm* 87, *emm* 94 and *emm* st1815) were recovered from children in both Brussels (15/20 *emm*-types, 84% of the isolates) and Brasília (15/48, 27% of the isolates).

#### New *emm* sub-types

8 new *emm* sub-types were identified. The 6 Brazilian new *emm* sub-types represented 7% of the isolates. They differed from the reference strains by a single nucleotide change. In Brussels, we found 2 new sub-types. One differed by a single substitution and the other by a 7 nucleotides change from the reference strains.

#### *emm*-types, *emm*-pattern, clinical presentation and site preference

**Figure 3:**
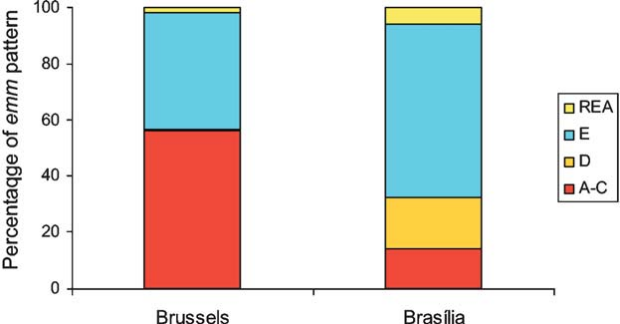
*emm* pattern of pharyngeal GAS in Brussels and Brasília. *emm* pattern A–C strains are throat specific, whereas *emm* pattern D strains have a skin tropism. *emm* pattern E strains are found at both sites. In Brussels, strains with *emm* patterns A–C (56%) and E (41.5%) were predominant among strains isolated from naso-pharynx (pharyngitis and scarlet fever included). In Brasília, the A–C pattern was rare (14%) amongst pharyngeal strains while E pattern (61.5%) and D pattern (18.5%) were predominant. REA: rearranged *emm* region.

*emm* pattern A–C strains are commonly found in the throat, whereas *emm* pattern D strains are most often isolated from skin lesions. *emm* pattern E is considered as “generalist”: It is readily found at both tissue sites. Among the Brazilian isolates, the relative risk for *emm* pattern A–C to be a throat isolate rather than a skin isolate was 1.68 (95% CI, 1.04–2.20). *emm* pattern D isolates had a relative risk to be recovered from skin rather than from throat of 2.16 (95% CI, 1.53–3.08). However, more *emm* pattern D than A–C were isolated from the throat of Brazilian children. A comparison of the *emm* pattern displayed by pharyngeal isolates recovered in both capitals is showed in Figure 3. The distribution of *emm*-pattern among Brazilian skin isolates showed a classical predominance of *emm* pattern D (55%) and E (42%) with few A–C (3%). In order to compare the *emm* pattern from the most relevant isolates from the two cities, the *emm*-types that were represented by a minimum of 3 isolates were selected (18/48 Brazilian *emm*-types and 16/20 Belgian ones). The proportion of these *emm*-types that was recovered from both cutaneous and pharyngeal sites was 66% (n = 12) in Brasília and 31% (n = 5) in Brussels. These *emm*-types were constituted mainly by *emm* pattern E (Brasília and Brussels, n = 7 and 3 respectively), but also *emm* pattern D (only in Brasília, n = 4) and A–C (in Brasília and Brussels, n = 1 and 2 respectively ).

The 2 cases of invasive infections in Brussels were caused by *emm* 1 and 3 strains belonging to the *emm* pattern A–C. By contrast, invasive infections in Brasília were associated with different *emm*-types (1, 49, 80, 87, st213 and st 6735), belonging to the three different *emm* pattern (E (n = 4), D (n = 1) and A–C (n = 1)).

## Discussion

This comparative study illustrates how GAS epidemiology features vary greatly in clinical and molecular aspects between two cities with different history, climate and socio-economical situation, although both performed in underprivileged populations attending public hospitals.

No GAS was isolated in children under 3 year-old in Brasília, while in Brussels, the GAS prevalence was similar in the 1 to 3 year-old group and in older children [14]. This contrasts with previous studies showing that the incidence of GAS pharyngitis was lower in children younger than 5 year-old than in older children [15]. Moreover, Belgian children with GAS pharyngitis were younger than Brazilian ones. Differences in day care attendance, administrated vaccines and antibiotics consumption in the first years of life might contributed to the variations we observed, as these factors can modify the naso-pharyngeal flora.

In the Belgian children, GAS infections were mostly attributable to *emm* pattern A–C and E strains. In Brasília, the *emm* pattern A–C was rarer than in Brussels while *emm* pattern D was much more frequent. The predominance of *emm* pattern D strains in tropical epidemiology has already been described [16]. However, the Brazilian *emm* pattern D strains, which classically have a skin tropism, presented interesting characteristics: (i) *emm* pattern D strains were recovered in one fifth (18.5%) of the isolates collected from the throat of Brazilian children. (ii) one third of the “non unique” Brazilian *emm*-types (which were represented by a minimum of 3 isolates) recovered from both throat and skin sites belonged to *emm* pattern D. (iii) *emm* pattern D strains (*emm* 53.0) were simultaneously isolated from the skin and the throat of two children presenting a sore throat associated with a skin infection. Some *emm* pattern D could therefore be considered as “generalist” with variable skin tropism. Similar findings were found in Ethiopia [7] and Nepal [9], where the *emm* pattern D strains were equally isolated from the skin and the throat. Previous studies showed that *emm* pattern D strains presented (with *emm* pattern E) the higher level of *emm*-type and multilocus sequence type (ST) diversity among the different *emm* pattern [10]. Recombination events appeared to be the most frequent in *emm* pattern D strains and included replacement of the *emm*-type locus [10]. Similarly, multilocus sequence typing identified substantial genetic recombination between the so-called skin and throat strains as well as various new combinations of *emm* and housekeeping genes in a small community with high rates of rheumatic fever and GAS impetigo [17]. Some *emm* pattern D strains might have evolved to acquire the capacity to colonize and/or infect both cutaneous and pharyngeal environment and therefore cause rheumatic fever. In Brasília, where rheumatic fever is endemic (Rheumatic fever was responsible of one third of the Brazilian heart surgeries in 2004; data from the Brazilian ministry of Health, Sistema Único de Saúde (SUS) - *Ministério da Saúde*), GAS skin infections were frequent and *emm* pattern D strains were more “generalist” than previously thought. Moreover, the A–C strains (classically associated with rheumatic fever) were rarely isolated. These findings are in agreement with the possible link between cutaneous strains and rheumatic fever in some epidemiological contexts. Indeed, if in temperate regions, rheumatic fever appears to be associated exclusively with throat infections, recent data suggested that GAS impetigo strains are probably involved directly or indirectly in acute rheumatic fever in particular communities [11], [16], [17]. In Ethiopia, *emm* pattern D strains were over represented (57%; 4/7) in the isolates associated with rheumatic fever [7]. Skin infections might constitute the GAS reservoir which triggers auto-immune reaction with or without secondary throat colonisation.

The high rate of rheumatic fever in Brasília could also be associated with the impressive *emm*-types diversity we observed (48 *emm*-types over 128 isolates). This wide variety of *emm*-type circulating in Brasília is probably responsible for a great diversity of anti-M proteine antibodies, hence increasing the risk for autoimmune response. Reports of GAS epidemiology outside Western nations are scarce but contradictory. Some of them (Korea, Mexico and aboriginal Australian) show the presence of a few predominant *emm*-types [11], [18], [19]. In other countries (India, Ethiopia and Nepal), a high diversity of *emm*-types was reported [6]–[9], although they were different from one location to the other. The studies showing a highly polyclonal distribution were performed in locations where massive and recent human migrations occurred. The city of Brasília was built in a poor deserted region 45 years ago. The population of Brasília, which has become one of the most developed city of the country, is now about 2 millions and immigrated from the whole country. The resulting diversity of hosts might contribute to the diversity of *emm*-types. By contrast with the Brazilian data, the *emm*-type distribution in Brussels was oligoclonal as typically seen in western countries [20].

This comparative study suggests that differences in the selective pressure between industrialised and low incomes regions of the world could favour the genetic evolution of GAS *emm*-types and therefore their emergence and/or persistence. Further studies are needed to clarify these differences and their impact for the prevention of GAS infections and sequelae. The theoretical coverage of the 26 valent GAS vaccine [21] would be 76% in Brussels whereas in Brasília, it would be as low as 32%. This low theoretical coverage is similar to that reported in the studies from India, Ethiopia and Nepal. This is of particular relevance as these countries are those who could most benefit from a GAS vaccine.

## Materials & Methods

We prospectively studied the epidemiology of GAS infections from February 1 to October 31, 2004 in children from Brussels and Brasília.

### Patients

Patients 0–15 year old attending 3 public hospitals of Brasília (Hospital Universitário de Brasília, Hospital Regional da Asa Sul and Unidade mixta de Saude de São Sebastião with 250, 180 and 40 beds respectively) and one public hospital of Brussels (Hôpital Universitaire des Enfants Reine Fabiola with 170 beds) were included in this study. This study was approved by the ethical board of all participant hospitals. An informed consent was obtained from each child's parent or guardian. Inclusion criteria were any infections with clinical suspicion of GAS aetiology or GAS isolation by the laboratory. Exclusion criterion was a current antimicrobial treatment. A pre-designed form was filled out by the pediatricians with personal, epidemiological and clinical data. GAS pharyngitis, cutaneous infections and otitis were defined as a positive GAS culture associated with symptoms of sore throat, impetigo and discharge from ear respectively. Scarlet fever was considered as clinically distinct from pharyngitis. Invasive infections were defined by a GAS isolation from a normally sterile body site. Post-streptococcal glomerulonephritis and acute rheumatic fever were defined according to published criteria [13]. Physicians were trained to swab with a standardised technique. No clinical follow-up was achieved.

### Collection and isolation

Samples were plated on blood agar (5%) and were incubated at 37°C for 24–48H in the presence of 5% CO_2_. Bloodcultures were incubated (Bactec®, Becton Dickinson, Frankin Lakes, United States) at 37°C for seven days. Beta haemolytic streptococci were phenotypicaly identified by beta haemolysis on blood agar, colony morphology, gram stain, catalase reaction and sensitivity to 0.04 U bacitracin disk. GAS identification was performed with a positive latex agglutination test containing group A specific antisera (Slidex; Biomérieux, Paris, France).

### *emm* typing

*emm*-typing of all isolates was performed according to the protocol described by the Center for Disease Control (CDC; http://www.cdc.gov/ncidod/biotech/strep/protocols.html). Primer 1 (5′-TAT TCG CTT AGA AAA TTA A-3′) and primer 2 (5′-GCA AGT TCT TCA GCT TGT TT-3′) or primer MF2 (5′-GGA TCC ATA AGG AGC ATA AAA ATG GCT A-3′) and MR1 (5′-TGA TAG CTT AGT TTT CTT CTT TGC GTT TT-3′) were used to amplify the 5′ region of the *emm* gene as described by the CDC. emmseq2 primer (5′-TAT TCG CTT AGA AAA TTA AAA ACA GG-3′) and MF2 were used for sequencing. The sequences were compared to those in the databases available on the CDC website (http://www.cdc.gov/ncidod/biotech/strep/strepblast.htm) and in GenBank (http://www.ncbi.nlm.nih.gov/BLAST/). New sequences were submitted to the CDC streptococcal database and to GenBank (accession number: *emm* 5.50, DQ 114470; *emm* 12.30, DQ 114469; *emm* st 2940.2, DQ 006844; *emm* 53.5, DQ 006846; *emm* 73.5, DQ 006845; *em*m 64.5, DQ 020479; *emm* 94.2, DQ 026516; *emm* 98.2, DQ 020480).

### *emm* pattern

GAS isolates within a given *emm* type usually display the same *emm* pattern [10].The *emm* pattern for each isolate was predicted according to McGregor et al., 2004 [10].

### Statistical Analysis:

Bilateral chi-square and student t test (SPSS 11.0, Gaussian distribution) were performed to compare general data and nutrition indices. p values<0.05 were considered significant.

## Supporting Information

Table S1 emm-types, sub-type and pattern by clinical presentation in Brussels.REA: rearranged, Empy: empyeme, TSS: toxic shock syndrome, RF: rheumatic fever, Vag: vaginal infection, Uri: urinar infection, Ocu: ocular infection.
(0.11 MB DOC)

Table S2 emm-types, sub-type and pattern by clinical presentation in Brasília.REA: rearranged, TSS: toxic shock syndrome, Cell: celulitis
(0.17 MB DOC)
