# Supplementary material for: Differences between Belgian and Brazilian Group A Streptococcus Epidemiologic Landscape
Source: PLoS One. 2006 Dec 20;1(1):e10. doi: 10.1371/journal.pone.0000010 (PMC1762354; doi:10.1371/journal.pone.0000010)
Supplement: Table S1 — emm-types, sub-type and pattern by clinical presentation in Brussels.REA: rearranged, Empy: empyeme, TSS: toxic shock syndrome, RF: rheumatic fever, Vag: vaginal infection, Uri: urinar infection, Ocu: ocular infection. (0.11 MB DOC) [file pone.0000010.s001.doc]

|  | *Emm* type | *Emm* sub-type | *emm* pattern | Number of isolates | % | Impetigo | Pharyngitis | Otitis | Scarlet  fever | Invasive inf | Sequelae | Others | Comments |
| --- | --- | --- | --- | --- | --- | --- | --- | --- | --- | --- | --- | --- | --- |
|  | *emm* 1 | *emm* 1.0 | A-C | 26 | 12.74 | 2 | 19 | 3 | 1 | 1 (Empy) |  |  |  |
|  |  | *emm* 1.29 | A-C | 1 | 0.49 |  | 1 |  |  |  |  |  |  |
|  | *emm* 2 | *emm* 2.0 | E | 3 | 1.47 |  | 2 |  |  |  |  | 1 (Vag) |  |
|  | *emm* 3 | *emm* 3.1 | A-C | 14 | 6.86 |  | 11 | 2 | 1 |  |  |  |  |
|  |  | *emm* 3.19 | A-C | 1 | 0.49 |  |  |  |  | 1 (TSS/RF) |  |  |  |
|  |  | *emm* 3.23 | A-C | 2 | 0.98 |  | 1 | 1 |  |  |  |  |  |
|  | *emm* 4 | *emm* 4.0 | E | 22 | 10.78 |  | 18 |  | 3 |  |  | 1 (Uri) |  |
|  | *emm* 5 | *emm* 5.46 | A-C | 2 | 0.98 |  | 2 |  |  |  |  |  |  |
|  |  | *emm* 5.50 | A-C | 1 | 0.49 |  | 1 |  |  |  |  |  | New sub-type |
|  | *emm* 6 | *emm* 6.0 | A-C | 22 | 10.78 |  | 18 | 4 |  |  |  |  |  |
|  |  | *emm* 6.14 | A-C | 1 | 0.49 |  | 1 |  |  |  |  |  |  |
|  |  | *emm* 6.4 | A-C | 13 | 6.37 |  | 12 | 1 |  |  |  |  |  |
|  |  | *emm* 6.5 | A-C | 4 | 1.96 |  | 3 | 1 |  |  |  |  |  |
|  | *emm* 9 | *emm* 9.0 | E | 4 | 1.96 |  | 3 |  | 1 |  |  |  |  |
|  | *emm* 11 | *emm* 11.0 | E | 6 | 2.94 | 1 | 5 |  |  |  |  |  |  |
|  |  | *emm* 11.1 | E | 1 | 0.49 |  | 1 |  |  |  |  |  |  |
|  | *emm* 12 | *emm* 12.0 | A-C | 21 | 10.29 | 1 | 19 | 1 |  |  |  |  |  |
|  |  | *emm* 12.13 | A-C | 1 | 0.49 |  | 1 |  |  |  |  |  |  |
|  |  | *emm* 12.30 | A-C | 1 | 0.49 |  | 1 |  |  |  |  |  | New sub-type |
|  | *emm* 28 | *emm* 28.0 | E | 9 | 4.41 |  | 8 | 1 |  |  |  |  |  |
|  | *emm* 44/61 | *emm* 44/61.0 | E | 4 | 1.96 |  | 4 |  |  |  |  |  |  |
|  | *emm* 58 | *emm* 58.0 | E | 1 | 0.49 |  | 1 |  |  |  |  |  |  |
|  | *emm* 64 | *emm* 64.0 | D | 1 | 0.49 |  |  | 1 |  |  |  |  |  |
|  | *emm* 75 | *emm* 75.0 | E | 3 | 1.47 | 1 | 2 |  |  |  |  |  |  |
|  | *emm* 78 | *emm* 78.0 | E | 3 | 1.47 |  | 3 |  |  |  |  |  |  |
|  | *emm* 87 | *emm* 87.0 | E | 4 | 1.96 |  | 4 |  |  |  |  |  |  |
|  | *emm* 89 | *emm* 89.0 | E | 22 | 10.78 | 2 | 19 |  |  |  |  | 1 (Ocu) |  |
|  | *emm* 94 | *emm* 94.0 | E | 2 | 0.98 |  | 2 |  |  |  |  |  |  |
|  | *emm* 102 | *emm* 102.3 | E | 1 | 0.49 |  | 1 |  |  |  |  |  |  |
|  | st 1815 | st 1815.0 | REA | 4 | 1.96 |  | 4 |  |  |  |  |  |  |
|  | Unknown | Unknown | Unknown | 4 | 1.96 |  | 4 |  |  |  |  |  |  |
| Total |  |  |  | 204 | 100 | 7 | 171 | 15 | 6 | 2 | 0 | 3 |  |

Table S1: emm-types, sub-type and pattern by clinical presentation in Brussels. REA: rearranged, Empy: empyeme, TSS: toxic shock syndrome, RF: rheumatic fever, Vag: vaginal infection, Uri: urinar infection, Ocu: ocular infection.
