# Supplementary material for: Differences between Belgian and Brazilian Group A Streptococcus Epidemiologic Landscape
Source: PLoS One. 2006 Dec 20;1(1):e10. doi: 10.1371/journal.pone.0000010 (PMC1762354; doi:10.1371/journal.pone.0000010)
Supplement: Table S2 — emm-types, sub-type and pattern by clinical presentation in Brasília.REA: rearranged, TSS: toxic shock syndrome, Cell: celulitis (0.17 MB DOC) [file pone.0000010.s002.doc]

|  | *emm* type | *emm* sub-type | *emm* pattern | Number of isolates | % | Impetigo | Pharyngitis | Otitis | Scarlet  fever | Invasive inf | Sequelae | Others | Comments |
| --- | --- | --- | --- | --- | --- | --- | --- | --- | --- | --- | --- | --- | --- |
|  | *emm* 1 | *emm* 1.0 | A-C | 3 | 2.30 | 1 | 1 |  |  | 1 (TSS) |  |  |  |
|  | *emm* 2 | *emm* 2.0 | E | 3 | 2.30 |  | 3 |  |  |  |  |  |  |
|  | *emm* 3 | *emm* 3.1 | A-C | 1 | 0.77 |  | 1 |  |  |  |  |  |  |
|  | *emm* 4 | *emm* 4.0 | E | 1 | 0.77 |  |  |  | 1 |  |  |  |  |
|  | *emm* 6 | *emm* 6.0 | A-C | 1 | 0.77 |  | 1 |  |  |  |  |  |  |
|  | *emm* 8 | *emm* 8.0 | E | 5 | 3.84 | 2 | 3 |  |  |  |  |  |  |
|  | *emm* 11 | *emm* 11.0 | E | 2 | 1.53 |  | 2 |  |  |  |  |  |  |
|  | *emm* 12 | *emm* 12.0 | A-C | 4 | 3.07 |  | 4 |  |  |  |  |  |  |
|  | *emm* 19 | *emm* 19.4 | A-C | 2 | 1.53 |  | 2 |  |  |  |  |  |  |
|  | *emm* 22 | *emm* 22.0 | E | 10 | 7.69 | 1 | 8 |  | 1 |  |  |  |  |
|  |  | *emm* 22.1 | E | 1 | 0.77 |  | 1 |  |  |  |  |  |  |
|  | *emm* 28 | *emm* 28.0 | E | 1 | 0.77 | 1 |  |  |  |  |  |  |  |
|  | *emm* 33 | *emm* 33.1 | D | 4 | 3.07 | 4 |  |  |  |  |  |  |  |
|  | *emm* 41 | *emm* 41.1 | D | 1 | 0.77 |  | 1 |  |  |  |  |  |  |
|  | *emm* 43 | *emm* 43.5 | D | 2 | 1.53 | 2 |  |  |  |  |  |  |  |
|  | *emm* 44/61 | *emm* 44/61.0 | E | 5 | 3.84 | 2 | 3 |  |  |  |  |  |  |
|  | *emm* 48 | *emm* 48.1 | E | 1 | 0.77 |  |  |  | 1 |  |  |  |  |
|  | *emm* 49 | *emm* 49.3 | E | 7 | 5.38 | 2 | 2 |  | 2 | 1 (Cell) |  |  |  |
|  | *emm* 53 | *emm* 53.0 | D | 9 | 6.92 | 5 | 4 |  |  |  |  |  |  |
|  |  | *emm* 53.5 | D | 1 | 0.77 |  | 1 |  |  |  |  |  | New sub-type |
|  | *emm* 58 | *emm* 58.0 | E | 6 | 4.61 | 3 | 3 |  |  |  |  |  |  |
|  | *emm* 59 | *emm* 59.0 | D | 5 | 3.84 | 3 | 1 |  | 1 |  |  |  |  |
|  | *emm* 60 | *emm* 60.1 | E | 1 | 0.77 |  | 1 |  |  |  |  |  |  |
|  | *emm* 63 | *emm* 63.0 | E | 1 | 0.77 | 1 |  |  |  |  |  |  |  |
|  | *emm* 64 | *emm* 64.5 | D | 1 | 0.77 | 1 |  |  |  |  |  |  | New sub-type |
|  | *emm* 66 | *emm* 66.0 | E | 1 | 0.77 | 1 |  |  |  |  |  |  |  |
|  | *emm* 73 | *emm* 73.0 | E | 1 | 0.77 | 1 |  |  |  |  |  |  |  |
|  |  | *emm* 73.5 | E | 1 | 0.77 | 1 |  |  |  |  |  |  | New sub-type |
|  | *emm* 74 | *emm* 74.0 | D | 3 | 2.30 | 2 | 1 |  |  |  |  |  |  |
|  | *emm* 75 | *emm* 75.0 | E | 1 | 0.77 |  | 1 |  |  |  |  |  |  |
|  | *emm* 76 | *emm* 76.0 | E | 1 | 0.77 |  | 1 |  |  |  |  |  |  |
|  | *emm* 80 | *emm* 80.1 | D | 3 | 2.30 | 1 | 1 |  |  | 1 (Cell) |  |  |  |
|  | *emm* 82 | *emm* 82.0 | E | 1 | 0.77 | 1 |  |  |  |  |  |  |  |
|  | *emm* 83 | *emm* 83.1 | D | 6 | 4.61 | 6 |  |  |  |  |  |  |  |

|  | *emm* type | *emm* sub-type | *emm* pattern | Number of isolates | % | Impetigo | Pharyngitis | Otitis | Scarlet  fever | Invasive inf | Sequelae | Others | Comments |
| --- | --- | --- | --- | --- | --- | --- | --- | --- | --- | --- | --- | --- | --- |
|  | *emm* 86 | *emm* 86.1 | D | 1 | 0.77 | 1 |  |  |  |  |  |  |  |
|  | *emm* 87 | *emm* 87.0 | E | 1 | 0.77 |  |  |  | 1 |  |  |  |  |
|  | *emm* 88 | *emm* 88.2 | E | 2 | 1.53 | 1 | 1 |  |  |  |  |  |  |
|  | *emm* 92 | *emm* 92.0 | E | 4 | 3.07 | 3 | 1 |  |  |  |  |  |  |
|  | *emm* 93 | *emm* 93.1 | D | 1 | 0.77 | 1 |  |  |  |  |  |  |  |
|  | *emm* 94 | *emm* 94.2 | E | 1 | 0.77 | 1 |  |  |  |  |  |  | New sub-type |
|  | *emm* 95 | *emm* 95.0 | D | 2 | 1.53 | 1 | 1 |  |  |  |  |  |  |
|  | *emm* 98 | *emm* 98.2 | D | 1 | 0.77 |  | 1 |  |  |  |  |  | New sub-type |
|  | *emm* 118 | *emm* 118.0 | E | 2 | 1.53 | 1 |  |  |  |  | 1 (AGN) |  |  |
|  | st 204 | st 204.0 | D | 1 | 0.77 | 1 |  |  |  |  |  |  |  |
|  | st 213 | St 213.0 | E | 3 | 2.30 |  | 1 |  | 1 | 1 (Cell) |  |  |  |
|  | st 1815 | st 1815.0 | REA | 4 | 3.07 |  | 4 |  |  |  |  |  |  |
|  | st 2904 | st 2904.1 | E | 1 | 0.77 | 1 |  |  |  |  |  |  |  |
|  | st 2911 | st 2911.0 | D | 1 | 0.77 | 1 |  |  |  |  |  |  |  |
|  | st 2940 | st 2940.2 | D | 4 | 3.07 | 4 |  |  |  |  |  |  | New sub-type |
|  | st 3765 | st 3765.0 | A-C | 1 | 0.77 | 1 |  |  |  |  |  |  |  |
|  | st 6735 | st 6735.0 | E | 2 | 1.53 |  | 1 |  |  | 1 (Cell) |  |  |  |
|  | Unknown | Unknown | Unknown | 2 | 1.53 |  | 1 |  | 1 |  |  |  |  |
| Total |  |  |  | 130 | 100 | 58 | 57 | 0 | 9 | 5 | 1 |  |  |

Table S2: emm-types, sub-type and pattern by clinical presentation in Brasília. REA: rearranged, TSS: toxic shock syndrome, Cell: celulitis
